# In Vivo Brain GSH: MRS Methods and Clinical Applications

**DOI:** 10.3390/antiox10091407

**Published:** 2021-09-01

**Authors:** Francesca Bottino, Martina Lucignani, Antonio Napolitano, Francesco Dellepiane, Emiliano Visconti, Maria Camilla Rossi Espagnet, Luca Pasquini

**Affiliations:** 1 Medical Physics Department, Bambino Gesù Children’s Hospital IRCCS, 00165 Rome, Italy; francesca.bottino@opbg.net (F.B.); martina.lucignani@opbg.net (M.L.); 2 Neuroradiology Unit, NESMOS Department, Sant’Andrea Hospital, La Sapienza University, 00189 Rome, Italy; francescodellepiane.us@gmail.com (F.D.); mcamilla.rossi@opbg.net (M.C.R.E.); luca.pasquini@uniroma1.it (L.P.); 3 Neuroradiology Unit, Surgery and Trauma Department, Maurizio Bufalini Hospital, 47521 Cesena, Italy; emiliano.visconti@gmail.com; 4 Neuroradiology Unit, Bambino Gesù Children’s Hospital IRCCS, 00165 Rome, Italy; 5 Neuroradiology Service, Department of Radiology, Memorial Sloan Kettering Cancer Center, New York, NY 10065, USA

**Keywords:** glutathione (GSH), magnetic resonance spectroscopy (MRS), neurological disorders

## Abstract

Glutathione (GSH) is an important antioxidant implicated in several physiological functions, including the oxidation−reduction reaction balance and brain antioxidant defense against endogenous and exogenous toxic agents. Altered brain GSH levels may reflect inflammatory processes associated with several neurologic disorders. An accurate and reliable estimation of cerebral GSH concentrations could give a clear and thorough understanding of its metabolism within the brain, thus providing a valuable benchmark for clinical applications. In this context, we aimed to provide an overview of the different magnetic resonance spectroscopy (MRS) technologies introduced for in vivo human brain GSH quantification both in healthy control (HC) volunteers and in subjects affected by different neurological disorders (e.g., brain tumors, and psychiatric and degenerative disorders). Additionally, we aimed to provide an exhaustive list of normal GSH concentrations within different brain areas. The definition of standard reference values for different brain areas could lead to a better interpretation of the altered GSH levels recorded in subjects with neurological disorders, with insights into the possible role of GSH as a biomarker and therapeutic target.

## 1. Introduction

Glutathione (GSH) is an antioxidant metabolite originating from glutamic acid (Glu), cysteine (Cys), and Glycine (Gly) amino acids, globally present in all mammalian cells [1]. Among its many roles, GSH is mainly implicated in oxidation−reduction reactions, acting as a protector against endogenous and exogenous toxic agents like reactive oxygen species (ROS) and reactive nitrogen species (RNS) [2]. Changes in the GSH brain concentration from oxidative stress may reflect inflammatory processes and mitochondrial dysfunction associated with biological aging [3] and pathological conditions [4,5]. In particular, as high levels of ROS may lead to cerebral tissue damage, the altered GSH concentration of specific brain areas has been described in several neurologic disorders, including epilepsy [6,7], multiple sclerosis [8,9], Alzheimer’s disease [10], Parkinson’s disease [11,12], and psychiatric disorders [13,14,15,16]. In order to provide a clear and thorough understating of GSH metabolism within the brain, an accurate and reliable estimation of cerebral concentrations needs to be performed. The quantification of GSH brain levels was first attempted ex vivo from autoptic specimens, by means of liquid chromatography with UV detection and spectroscopic techniques [17,18,19]. GSH biosynthesis and metabolism were also tested in vitro, where different cell culture models were investigated to assess oxidative stress levels from blood and cerebrospinal fluids [20,21]. More recently, in vivo GSH measurements were obtained using proton magnetic resonance spectroscopy (MRS), a powerful non-invasive technique for brain metabolite quantification. Although widely used for GSH detection in both animals and humans [22], MRS presents several technical challenges, mostly related to the low GSH brain concentration and severe spectral overlapping between metabolites with different peak intensities [23]. Many MRS techniques have been developed for GSH concentration assessment, with a high methodologic heterogeneity, which may limit a comparative evaluation of the results provided by different studies. For this reason, the literature is still lacking a comprehensive and detailed description of the GSH normal levels within different specific brain areas. This information appears crucial for the interpretation of GSH findings in the normal brain and neurologic disorders, providing a valuable benchmark for clinical applications. In this context, the objective of this work was to describe the various MRS techniques available today for in vivo human brain GSH quantification, providing an overview of the different methodologies and applications, including an exhaustive list of normal GSH concentrations within different brain areas (e.g., the amygdala and anterior cingulate cortex). Moreover, we reviewed the participation of GSH in brain disorders, in order to gain insight into its possible role as a biomarker and therapeutic target.

## 2. GSH Metabolism

**Figure 1:**
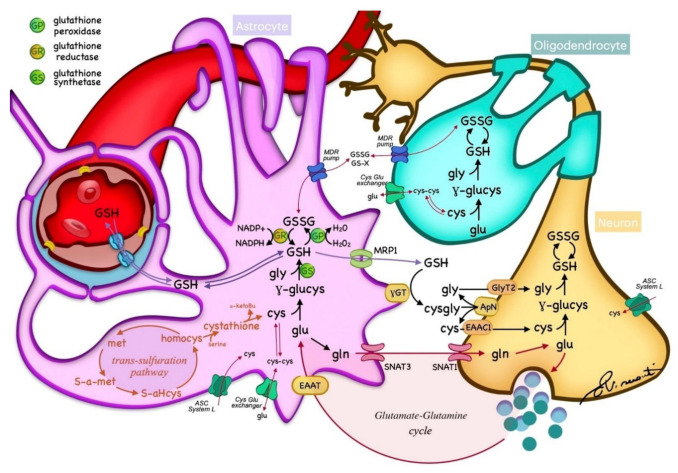
Glutathione (GSH) metabolism within the nervous tissue. GSH is synthesized in the cytoplasm of neurons and glia from essential amino acids, and catabolized through hydrolysis in the cell membranes. GSH acts as a reducing agent by donating an electron to H_2_O_2_, leading to the formation of H_2_O, O_2_, and glutathione disulfide (GSSG), which is regenerated by glutathione reductase (GR) from NADPH. The transportation of GSH and essential metabolites is regulated by different transporters across cell membranes. Cys—cysteine; glu—glutamate; gln—glycine; met—methionine; homocys—homocysteine; MPR—multidrug resistance pump; γGT—γ-glutamyltransferase; γ-glucys—γ-glutamylcysteine; EAAT—excitatory amino acid transporter; SNAT—sodium-coupled neutral amino acid transporter; ASC—alanine, serine, and cysteine transport system.

GSH is abundant in the brain, with a high concentration in non-neuronal cells, mostly neuropil and white matter tracts, with the exception of some cerebellar neurons, such as granule cells and Purkinje cells [22]. Within the brain, GSH is synthesized from the essential amino acids Glu, Cys, and Gly in a two-step reaction catalyzed by ATP-dependent enzymes. In the first step, Glu is combined with Cys by γ-glutamylcysteine synthetase (or glutamate−cysteine ligase (GCL) EC 6.3.2.2) to form γ-Glu−Cys. This dipeptide is further combined with Gly by glutathione synthetase (GS; EC 6.3.2.3) to produce GSH [1]. GSH catabolism is realized through hydrolysis by γ-glutamyltransferase (γGT; EC 2.3.2.2), which is located in the cell membranes of many cells throughout the body. In the brain, γGT is located in non-neuronal cells, mostly ependymal cells, and secondarily in Schwann and glial cells [22]. GSH metabolism is summarized in Figure 1. GSH fulfills its antioxidant role through two main mechanisms: (1) direct non-enzymatic reaction with free radicals such as superoxide (O_2_^−^), NO, or hydroxide (OH^−^), and by (2) acting as a reducing agent in the presence of glutathione peroxidase (GP), by donating an electron to H_2_O_2_, leading to the formation of H_2_O, O_2_, and glutathione disulfide (GSSG) [1]. In turn, glutathione reductase (GR) regenerates GSH by transferring an electron from NADPH to GSSG (Figure 1). This enzyme is mostly expressed in oligodendrocytes, microglia, and neurons, with a lower expression in astrocytes [22]. Another major role of GSH is the detoxification and removal of xenobiotics and other endogenous compounds, that are conjugated with GSH by glutathione-S-transferase to be exported from the cell through multidrug resistance pumps (MRPs), the main GSH transporters [22,24]. Furthermore, GSH is a cofactor of various enzymes. For example, the glyoxalase enzyme system catalyzes the detoxification of ketoaldehyde methylglyoxal (a very reactive molecule that mediates protein denaturation) to D-lactate with the participation of GSH [22].

## 3. Noninvasive GSH Measurement

GSH can be non-invasively assessed using MRS. Although the feasibility of measuring GSH with MRS has already been demonstrated [25], it is still difficult to translate this procedure into clinical practice because of the low GSH concentration in the brain (1.5–3 mmol/L), low signal-to-noise ratio (SNR) of the brain spectra, and severe spectral overlapping between metabolites with different peak intensities [23]. Moreover, several aspects need to be considered when using MRS for GSH assessment, including the magnetic field homogeneity required for spectral acquisition, water and lipid suppression for accurate metabolite detection, as well as the intrinsic complexity of spectral analyses [26]. For these reasons, during recent years, several methods have been proposed to assess GSH concentration in vivo within the human brain, trying to mitigate the aforementioned problems. Firstly, to better detect low-concentration metabolites such as GSH, the water peak of the spectrum needs to be suppressed with an appropriate frequency-selective water suppression routine [22]. Among the available methods for water and lipid suppression, the most used is the chemical shift selective saturation (CHESS) and its relative variants (e.g., variable power radio frequency pulses with optimized relaxation delays (VAPOR)) [27,28,29]. The MRS approach requires localizing the MR signal within a specific brain region, either by the exiting signal in a single rectangular volume of tissue (i.e., single voxels spectroscopy (SVS)) or by using gradients for spatial encoding over a large volume of tissue (i.e., multiple voxel shift imaging (CSI or MRSI)). SVS techniques are the most widely used as they provide high-quality spectra with excellent shimming and high SNR [30,31]. On the other hand, multi-voxel CSI represents a suitable solution when large and heterogeneous brain areas need to be investigated, as it allows for acquiring a larger area with a higher spatial resolution at the cost of longer scan times, lower SNR, and possible spectral contamination from adjacent voxels [32]. Conventional approaches for GSH detection require the acquisition of a short echo (TE = 5–30 ms) localized spectrum to reduce signal decay related to transverse relaxation [23]. In this context, “first generation” methods consisted of the acquisition of a localized spectrum (i.e., unedited spectrum), from which GSH was quantified with a least squares fitting based on an “a priori” metabolite model [33]. Although widely used in clinical practice [22], the fitting of unedited spectra provides ambiguous GSH quantification, mostly dependent on spectral quality and baseline [34]. In fact, the metabolite signal for the short TE is always superimposed on the baseline spectral produced by macromolecules (MM), which, in turn are responsible for fitting the performance degradation [34,35]. In order to provide unambiguous detection of small metabolites, spectral editing techniques were introduced as “second generation” methods for GSH quantification. These approaches depend on longer TEs (TE = 70–130 ms) and exploit J-coupling between spins to reduce overlapping issues and better discriminate between metabolites, but lead to increased sensitivity to patient motion and instrumental instabilities [36,37]. Alternative approaches were also introduced to overcome metabolite overlap, including more advanced shimming technologies [23], spreading out the signal into a second frequency dimension (i.e., 2D MRS), or the use of higher B0, as the relative width of multiplets (in ppm) is inversely proportional to the field strength [37]. Given the pivotal role of GSH in the human brain, an increasing number of studies have been performed with sophisticated MRS techniques to reliably assess GSH concentration [22,38]. Although the methods reported in the literature are highly heterogeneous (i.e., different acquisition techniques, different voxel size and placement, and different post-processing), we provide a detailed description of the current methods for GSH measurement within the next sections, differentiating unedited and edited spectrum techniques. For each of the studies included in this review, we specify the number of participants enrolled, the acquisition techniques and characteristics, eventual data-processing tools, and the brain areas analyzed together with their relative GSH levels.

### 3.1. GSH Measurement—Unedited Techniques

Unedited techniques are non-selective methods able to provide complete localized spectra from which a series of metabolite are quantified by fitting the signal to an a priori metabolite model. Among the unedited techniques, single-voxel methods usually use three orthogonal slice-selective RF-pulses to detect a signal within a specific volume of interest (VOI), while signals outside the VOI are removed with specific field gradient pulses [39]. Commonly used and widely available unedited methods include point resolved spectroscopy (PRESS), stimulated echo acquisition mode (STEAM), and spin echo full intensity acquired localized (SPECIAL). All of these spectroscopic techniques are characterized by short TEs (5–30 ms) [22] that are responsible for minimizing the spectral multiple distortion and the transverse relaxation effect [40]. Although intrinsically sensitive to B1 variation, the PRESS sequence was implemented for GSH detection within the human brain for a variety of disorders [12,41,42,43,44] and at different magnetic field strengths (i.e., 1.5 T, 3 T, and 7 T) [45,46,47,48]. The frequency-selective RF pulses were also responsible for a dephasing of unwanted signals, requiring significant gradient spoiling that, in turn, increases the minimum TE for PRESS acquisitions when compared with the other unedited techniques like STEAM and SPECIAL [40]. In fact, the STEAM sequence provides efficient suppression of unwanted coherence pathways, producing high quality spectra with very short TEs (5–10 ms) [9]. The larger bandwidth of RF pulses in combination with STEAM provides a “stimulated echo”, making this technique less prone to chemical shift displacement errors [40]. Although the very short TEs made STEAM a suitable solution for clinical practice with applications of GSH detection in infants [49] and adults [50,51], this technique suffers from a two-fold signal loss compared with PRESS [46]. Moreover, the spin-echo-based PRESS sequence has twice the signal compared with STEAM, and is therefore often preferred for clinical applications [39].

SPECIAL combines the advantage of the short TEs typical of STEAM with the full signal intensity achieved with SE-based scans like PRESS [40]. This technique relies on a hybrid sequence pulse, able to provide excellent frequency selectivity and is responsible for shortening the minimum TE. Moreover, in order to overcome signal loss related to B1 inhomogeneity at a high field strength (e.g., 3 T or higher), the specific localization by adiabatic selective refocusing (LASER) technique can be implemented, together with its simplified version (i.e., semi-LASER) [52,53]. Both the LASER and semi-LASER sequences help at reducing the chemical shift displacement errors at the cost of a higher RF power requirement and longer TE compared with the conventional localization sequences [39]. Despite several reports of SPECIAL and semi-LASER applications for GSH detection in the healthy brain at different field strengths (e.g., 3 T and 7 T) [54,55,56], these techniques are not as commonly used as PRESS and STEAM.

Other approaches for reliable GSH detection include two-dimensional MRS techniques, where the directly acquired dimension contains both chemical shift and coupling information, while the indirectly acquired dimension only contains coupling information [37]. Thanks to its ability to resolve more metabolites than conventional 1D MRS, localized correlated spectroscopy (L-COSY) [57,58] has recently been proposed to assess the GSH brain concentration. Unfortunately, although a non-uniformly weighted sampling (NUWS) scheme was recently implemented to accelerate the acquisition [59], the long scanning time and technical challenges related to spectral artifacts have prevented the routine implementation of L-COSY in clinical practice [22]. The above-mentioned unedited techniques require specific post-processing routines (fitting) to provide metabolite quantification. Among the available tools for metabolite quantification (e.g., jMRUI and ProFit), one of the most widely used methods for spectral quantitation is the linear combination model (LCModel) [60]. LCModel is a well-established method that computes the best linear combination of basis-set that fits the acquired data, including automatic phase adjustment, frequency alignment, baseline subtraction, and eddy current correction [22,39]. The program returns metabolite concentrations (relative to an unsuppressed water signal), the fit, the residuals, and the uncertainty estimation (in terms of Cramer–Rao lower bounds (CRLB)). As CRLB is dependent on image SNR and is strictly applicable only if the model is correct and fully parametrized, a threshold of 20% is usually used to accept or reject metabolite quantification [61,62]. LCModel is commercially available and depends on prior knowledge of the individual metabolite spectra with the acquisition parameters used (a basis set) to fit the edited signals.

### 3.2. GSH Measurement—Edited Techniques

In brain MRS, the GSH signal is almost obscured by those from other metabolites that are present at much higher concentrations. Spectral editing techniques are able to detect GSH with the suppression of unwanted metabolite signals, allowing for a reduction of the spectrum complexity [63]. Conversely, compared with the unedited techniques, the edited techniques eliminate overlapping singlet resonances, providing a more unambiguous measure of GSH [35]. GSH edited techniques include multiple quantum filters (MQF) [25,64] and J-differences spectroscopy [65].

#### 3.2.1. Multiple Quantum Filter

Multiple quantum filter (MQF) can be considered a black-box mechanism for separating signals from a coupled spin system of interest from stronger overlying signals from other metabolites [63]. This technique removes unwanted signals experimentally from the spectrum within each repetition time. An advantage of MQF is the low sensibility to subject motion and scanner instability. MQF has been used in vivo at 3 T [8,35,66]. The MQF technique has the following two particular disadvantages: (i) the absence of a reference signal preserved by an MQF experiment, making quantification challenging, and (ii) the sensitivity of the experiment may be reduced due to the loss of signal [63]. Moreover, MQF requires in-house tools for analysis, limiting the widespread implementation of these methods [63]. For these reasons, most recent studies preferred the J-difference editing technique [10,67,68,69].

#### 3.2.2. J-Difference Editing

J-difference editing is often implemented through the MEGA-PRESS technique [28]. It is based on two sets of sub-experiments: the ON experiment, in which frequency selective editing pulses are applied to GSH spins, and the OFF experiment, in which these pulses are not applied (or are applied at another frequency) [70,71].

The metabolite signal is resolved with the subtraction of ON and OFF scans, resulting in a difference-edited spectrum where the signals of any metabolite that are unaffected by the editing pulses are removed [72]. Several studies have used phantoms data to provide evidence of the robust detection of the GSH volume in the human brain through this technique [65,67,73,74,75]. Sequence optimizations have been applied to simplify the GSH spectrum [76]. In this context, the choice of the optimum echo time for GSH MEGA-PRESS has been analyzed, and different studies have documented that TE ~120 ms is optimal both in pathological [77] and HC [76] subjects. The most widely used techniques for the post-processing of edited GSH spectra include in-house developed software (often in MATLAB environment), LCModel, and Gannet. One of the disadvantages of the LCModel is that it requires high-quality spectra and a reliable set of base spectra to minimize fitting errors. Gannet consist in a MATLAB-based open source software [78]. It is developed specifically for edited spectra and uses a simple Gaussian for fitting. As the MEGA-PRESS spectrum is the result of the subtraction of two repetition times, a disadvantage of the MEGA-PRESS method is the high sensibility to subject motion. Frequency and phase correction (FPC) approaches have allowed for overcoming this issue [72,79,80]. Recently adapted MEGA-PRESS sequences have been developed to reduce the acquisition time in studies based on the acquisition of more than one metabolite (e.g., GSH and GABA) or based on the acquisition of more than a voxel (e.g., a voxel in each hemisphere). In particular, multiplexed edited detection allows for GSH editing simultaneously with others metabolites in a single acquisition. Additionally, accelerated MEGA PRESS with parallel reconstruction in multivoxel simultaneously acquired J-difference-edited GSH spectra (MEGA-PRIAM) from two voxels.

##### Multiplexed Edited Detection

Multiplexed edited detection (achieved by adapting MEGA-PRESS) allows for editing GSH simultaneously with others metabolites in a single acquisition and includes double editing for ascorbate and GSH [3,81,82,83], and Hadamard encoding and reconstruction of MEGA-edited spectroscopy (HERMES) for GSH and GABA [84,85]. Saleh et al. proved that the quality and signal-to-noise ratio (SNR) of the GSH and GABA spectra obtained with HERMES were similar to those of the sequentially acquired MEGA-PRESS spectra, with the benefit of saving half the acquisition time [84]. Muhammed et al. developed a universal MEGA-PRESS sequence with HERMES functionality for the major MR vendor platforms with standardized RF pulse shapes, durations, amplitudes, and timings, allowing for the detection of both GABA- and GSH-edited spectra with a strong agreement among vendors [86]. Recently, a motion compensation technique was developed to reduce the amount of artifacts in the resulting edited spectra [76]. However, Marsman et al. investigated motion sensitivity in edited MRS (HERMES), analyzing the GABA and GSH spectra obtained both without motion (retrospective), with corrected motion (prospective with post-processing) and with uncorrected motion. In this study, the HERMES spectral editing data were shown to be sensitive to motion, as significant differences in metabolite estimates and variability of the spectral quality measures were observed for GSH when only the retrospective outlier removal was applied [87].

##### Accelerated MEGA PRESS with Parallel Reconstruction in Multivoxel

Oeltzscher et al. demonstrated that the simultaneous acquisition of J-difference-edited GSH spectra (MEGA-PRIAM) from two voxels was feasible at 3 T, finding that there was no significant difference between MEGA-PRIAM and single-voxel estimates of GSH. MEGA-PRIAM increased the data acquisition rates compared with MEGA-PRESS by a factor of two [75]. Saleh et al. provided both GABA and GSH measurements from two brain regions in a single scan using a combination of HERMES and MEGA-PRIAM [84].

### 3.3. GSH Measurement at High Fields

In order to mitigate the spectral overlapping issues for low-concentration metabolites such as GSH, high field acquisitions (i.e., 7 T) were recently used with both edited [77] and unedited sequences [3,47,48,88,89]. In addition to the higher SNR provided, high field acquisitions allowed for overcoming the metabolite-overlap issue, as the relative width of multiplets (in ppm) was inversely proportional to field strength [37].

## 4. Detection Techniques Reliability

Several studies have investigated and compared GSH detection reliability of MRS techniques [9,90,91,92]. In a recent study, Witenburg compared the reproducibility of STEAM, PRESS, SPECIAL, and MEGA-PRESS, showing the best reproducibility for STEAM, followed closely by PRESS, SPECIAL, and finally MEGA-PRESS [91]. In particular, Witenburg demonstrated reproducibility between the phantom concentration and detected GSH concentration using STEAM with a very low TE (i.e., 6.5 ms). However, as Witemburg did not use adjustment for optimal editing in the MEGA-PRESS sequence [93], the performance of MEGA-PRESS may have been underestimated in this study. Additionally, GSH reliability estimates using MEGA-PRESS sequence with the optimal TE (120 ms) were comparable to those obtained with PRESS in the study of Prescot [92,93]. Although widely used, the PRESS sequence seems to not be able to reliably detect the GSH concentration below 3 mM [82,94], producing an LCModel with a significantly non-zero concentration of GSH (1–2 mM) when acquiring phantoms that did not contain the metabolite [14,51,61]. Different to the unedited technique, MEGA-PRESS showed a good reliability detecting GSH concentrations over the full physiological range of 0–24 mM [22,82,94]. Consequently, J-difference editing using TE = 120 ms may be the best available sequence for measuring GSH, as it provides reliability estimates very similar to low TE STEAM and PRESS sequences, but, unlike those sequences, correctly detects the absence of GSH, avoiding false positives. Recently, MEGA-PRESS adapted sequences were implemented to reduce acquisition time, including MEGA-PRIAM and HERMES. As a study demonstrated that there was no difference between MEGA-PRIAM and single-voxel estimates of GSH, the MEGA-PRIAM sequence could be used to simultaneously acquire J-difference-edited GSH spectra from two voxels [75]. Prisciandaro showed that MEGA-PRESS provides more reproducible GSH values compared with HERMES, thus suggesting that despite HERMES providing a reasonable GSH concentration, MEGA-PRESS should be used when GSH measurements are of primary importance to the research question [93].

## 5. Brain Areas GSH Concentration

The acquisition sequence used for a specific study is a decisive step, as the GSH concentration and differences between groups could be different when selecting edited or unedited techniques. Dhamala showed strongly correlated GSH measures between SPECIAL and PRESS techniques, while a weak correlation occurred between MEGA-PRESS and both SPECIAL and PRESS [56]. Similarly, Nezhad reported a significant difference in GSH concentration estimates when comparing MEGA-PRESS with PRESS [94]. Moreover, this study showed more sensibility in edited (MEGA-PRESS) compared with unedited sequences (PRESS) when identifying differences between two brain area concentrations (i.e., anterior cingulate cortex and occipital cortex) only with MEGA-PRESS. As GSH detection has the potential to provide a better understanding of the oxidation−reduction balance in the human brain, several examples of both edited and unedited techniques have been reported in the literature, where VOI were placed in different brain areas, with sizes ranging from 15 mm^3^ [55] to 30 cm^3^ [9,35]. A comprehensive description of the GSH detection studies has been reported. We reported GSH concentration within the different brain areas investigated for HC subjects found in the literature (Table 1). Particularly, Table 1 reports the number of HC participants and the corresponding mean age, together with the main evidence found for each study. The definition of standard reference GSH values within the different brain areas reported could lead to a better interpretation of the altered GSH levels recorded in subjects with neurological disorders, with insight into the possible role of GSH as a biomarker and therapeutic target. Referring to the reliability previously discussed and the sensibility of MEGA-PRESS, the most reliable GSH detected values were those of studies that used this technique in brain area analysis through a comparison between groups and in clinical applications [3,10,67,74,77,83,95,96,97,98,99,100].

## 6. Clinical Applications of GSH Imaging

### 6.1. GSH and Brain Tumors

A decrease in GSH levels or the GSH/glutathione disulphide (GSSG) ratio promotes oxidative stress, leading to the progression of cancer. On the other hand, elevation of GSH levels enhance the antioxidant capacity of the cell and the resistance to oxidative stress, which may mediate treatment failure in cancer [100]. GSH cellular concentration is linked to the apoptotic process by interacting with caspase enzymes and transcription factors, ceramide production, thiol-redox signaling, and phosphatidylserine externalization [101,102]. GSH is also involved in a very specific cell death pathway by the name of ferroptosis [103]. To summarize, cancer cells are characterized by a peculiar redox microenvironment, where enhanced oxidative stress is accompanied by an increase in glutathione levels, leading to growth advantage and resistance to chemotherapeutic agents [104]. For example, a direct correlation between glutathione-S-transferases (GST) expression and anti-cancer drug resistance has been demonstrated [105,106], including through bioptic evidence from tumor tissues that gained drug therapy resistance [107,108]. As a consequence, the increased expression of GST often translates to a poor prognosis in gliomas [109,110,111,112].

The GSH concentration has been studied as a biomarker in different tumors, showing interesting correlations with molecular features. In the brain glioma, the mutation of IDH1 has a great impact on survival, and influences the cytoarchitecture and imaging appearance of the tumor [113,114]. This mutation seems to disrupt the NADP/NADPH balance, with an increased demand for glutathione. In addition, the nuclear factor erythroid 2–related factor 2 acts as a neuroprotector in IDH1-mutated cells by promoting GSH synthesis and scavenging reactive oxygen species [115]. Bisdas et al. observed significantly decreased GSH levels (39%, *p* = 0.019) in IDH mutant gliomas through MRS at 9.4 T, possibly caused by the depletion of NADPH during cancerogenesis [116]. Batsios et al. evaluated GSH-related metabolism through hyperpolarized MRI in mice. The authors detected higher levels of [1-13C] glycine in tumor-bearing rats compared with controls, and in tumor tissue compared with the normal brain. Higher [1-13C] glycine was accompanied by an enhanced GGT expression and increased GSH levels in the tumor tissue [117]. Other authors demonstrated that the mutation of IDH1 inhibits the growth of glioma cells, possibly mediating prolonged survival in the glioma. IDH mutant glioma cells seem to be characterized by the depletion of GSH and the generation of ROS [118]. Opstad et al. studied GSH levels in the meningioma through in vivo MRS at 1.5 T. The analysis of short echo time brain tumor spectra using a linear combination model highlighted a significant contribution of glutathione to the spectra, with a concentration of 3.3 ± 1.5 mM [119]. To conclude, more studies are necessary to investigate GSH metabolism in brain tumors and to assess the potential correlations with molecular biomarkers. The ability to noninvasively quantify GSH in vivo may improve the selection of tailored therapies, provide an indicator of tumor aggressiveness, and help prognostication.

### 6.2. GSH and Psychiatric Disorders

Imbalance of oxidative stress metabolites and atypical levels of glutathione (GSH) in specific areas of the brain have been reported in several psychiatric disorders such as schizophrenia, bipolar spectrum disorder, and depression, although no consistent results have emerged, possibly due to limited sample sizes [120]. One of the main efforts for GSH assessment with MRS in psychiatric disorders was published by the same group investigating GSH levels in depression [15], alcohol and tobacco abuse in bipolar disorder [14,121], and mood disorders with increased risk of psychosis [61]. This work led to the conclusion that GSH imbalance (both increased and decreased) is involved in the pathogenesis of these conditions.

Schizophrenia is the most studied disorder overall, although it is difficult to draw definite conclusions about the role of GSH as a biomarker of the disease, as different studies investigated different areas of the brain or used different scanners (1.5 T, 3 T, and recently 7 T) and different acquisition techniques [22]. Moreover, patients were investigated in different situations such as in early or stabilized disease, or with treated or drug-free subjects. A recent meta-analysis of previous papers demonstrated that small but significantly reduced levels of GSH in the anterior cingulate cortex (ACC) are related to psychotic manifestations in patients with schizophrenia [120]. More recently, GSH levels in schizophrenia were investigated using ultra-high field 7 T MRS. Kumar et al. found significantly lower levels of GSH in the ACC, left insula, and visual cortex of patients with stable schizophrenia (mean concentration of 1.55 ± 0.26, 1.68 ± 0.26, and 1.47 ± 0.20 mM, respectively) [24]; a recent meta-analysis of 255 patients with psychosis (121 first episode) confirmed the significantly lower brain GSH levels compared with HC by 7 T MRS [122]. GSH levels may also predict treatment response, as recently demonstrated in a study by Dempster et al., where higher GHS levels were correlated to a better response to drug therapy in patients with a first episode of psychosis [123]. Although this connection is well established, it is still debated whether dysregulation of GSH predisposes the development of psychosis in high-risk populations [95,124]. Recently, a study measured the GSH levels (GSH/H_2_O ratio between 0.0015–0.0018) in the prefrontal cortex of patients at high risk for psychosis, and no significant difference was found compared with HC [95]. However, another study by Demro et al. on a population of 12 adolescents found higher levels of GSH in the ACC and striatum related to positive symptoms such as grandiosity and disorganized communication (with a mean GSH concentration of 2.25 ± 0.42 mM in the ACC and 1.93 ± 0.54 mM in the striatum) [125]. Other authors have suggested that oxidative stress and abnormal levels of GSH in the brain are involved in early psychosis development. A recent study by Reyes-Madrigal et al. investigated striatal GSH and found decreased levels in patients with first-episode psychosis compared with the controls (mean GSH concentration 0.92 ± 0.24 × 10^−3^ mM) [98]. On the other hand, increased GSH levels in the medial temporal cortex were found to be related to first-episode psychosis by Wood et al. [126]. This finding is apparently in contrast with other evidence from the literature, maybe suggesting a compensatory response in the early stage of the illness. Oxidative stress and GSH imbalance have been related to other psychiatric conditions such as bipolar disorder [120], obsessive-compulsive disorder (OCD) [127], and post-traumatic stress disorder (PTSD) [97]. Autism spectrum disorder (ASD) is another condition in which oxidative stress-related damage has been proposed as a pathophysiological contributor. Several studies have demonstrated alterations in oxidation markers including GSH in affected patients, compared with HC. However, almost all of these studies were carried out with indirect measurements of GSH in the blood or post-mortem [128]. More recent studies with 3 T MRS through the in vivo evaluation of GHS levels found no significant difference in GSH levels between patients with ASD and the controls (Durieux et al. mean concentration 2.5 mM in the dorsomedial prefrontal cortex and 2.8 mM in the basal ganglia of both ASD patients and HC) [99,128].

Even if more research is needed, GSH levels offer a potentially valid biomarker to aid in the diagnosis of patients with Schizophrenia and other psychiatric conditions. Future developments will possibly include GSH-targeting drugs [129] and proton MRS to assess treatment selection and response.

### 6.3. GSH and Degenerative Disorders

It is recognized that oxidative stress plays a role in normal aging. Increased oxidative stress and lower GSH levels have been investigated as important pathogenic contributors in several age-related conditions, including ocular diseases (nuclear cataract, glaucoma, and macular degeneration), hearing impairment, and osteoporosis [101]. Nevertheless, a direct connection between brain glutathione metabolism and increasing age is still controversial. In a recent post-mortem study by Tong et al., no significant difference was found between GSH levels in the brains of healthy subjects of different ages [130]. On the other hand, GSH and redox imbalance seem to have a role in degenerative diseases of the CNS. Increased oxidative stress and a higher production of oxygen radicals in the mitochondria have been proposed as one of the main pathogenetic mechanisms in Alzheimer’s, Parkinson’s, and Huntington’s diseases [24,101]. Decreased levels of GSH and increased oxidative stress are related to a greater level of beta-amyloid in the brain, suggesting GSH as a possible biomarker of early Alzheimer’s disease (AD) [131]. Saharan et al. analyzed several prior studies on the correlation between GSH, AD progression, and cognitive decline, particularly focusing on blood levels of GSH. Brain GSH levels assessed by MRS were considered a promising tool for the diagnosis of AD [132]. Additionally, decreased GSH has been reported to precede the onset of amyloid plaques in mouse transgenic Alzheimer’s models [133], and higher levels of glutathione were measured in vivo in the brain of healthy subjects compared with AD patients [96]. Recently, Duffy et al. reported mild cognitive impairment being associated with increased GSH levels in the anterior and posterior cingulate, with related effects on the neuropsychological performance (mean GSH concentration 0.47 ± 0.15 and 0.37 ± 0.07 mM) [42]. This finding is apparently in contrast with previous evidence; however, it may suggest an early compensatory response mechanism to increased oxidative stress during the onset of AD [129].

GSH metabolism has also been investigated in correlation with Parkinson’s disease (PD). However, although its role in pathogenesis is well established [24,101], the current evidence on GSH MRS assessment in PD patients is still scarce. Energetic metabolism impairment and oxidative stress have an important role in the onset and progression of amyotrophic lateral sclerosis (ALS), contributing to cellular damage and, eventually, to neuronal death. There is important evidence on elevated markers of oxidative damage in the tissues of ALS patients, including cerebrospinal fluid (CSF), spinal cord, and brain cortex [134]. Additionally, mutations in the antioxidant enzyme superoxide dismutase 1 (SOD1) have been implicated in about 20% of familial ALS cases [135]. Although conventional MRI is usually unremarkable in ASL, MRS can highlight dysregulation of the brain metabolites in the motor cortex of affected patients [136]. The specific levels of GSH were assessed in two studies by Weiduschat and Cheong [67,68], with no definitive results. In the first study, lower levels of GSH were measured in the motor cortex of affected patients compared with healthy volunteers (GSH/water ratio 1.1 ± 0.3 × 10^−3^ and GSH/total creatine ratio 1.2 ± 0.5 × 10^−2^) [67]. On the other hand, although metabolite impairment was recognized at 3 T MRS by Cheong et al., the GSH levels in ASL patients were comparable to HC (GSH concentration 1.0 µmol/g) [68].

### 6.4. OTHERS

- GSH and Epilepsy

Few studies in the literature have assessed the role of GSH and oxidative stress in epilepsy. It is widely suspected that epileptic seizures are accompanied by a high production of reactive oxygen species, increasing oxidative stress. This theory is supported by several studies in the literature, including recent evidence of increased levels of brain GSH in epileptic patients after ketogenic diet, a well-known adjuvant therapy in epilepsy [44]. In a 2001 study, GSH levels were measured with H-MRS in the parieto-occipital region of both hemispheres in patients with and without active epilepsy compared with controls. A significative decrease in the GSH/water ratio was found in patients with epilepsy compared with healthy subjects (GSH/water ratio of 1.6 ± 1.0 × 10^−5^ and 2.4 ± 1.1 × 10^−5^ mM, respectively) [137]. No difference was found in the GSH/water ratio between the two hemispheres of affected patients; this finding seems to suggest a widespread impairment of the glutathione system in patients with epilepsy, independently from the location of the epileptogenic focus. Similarly, a more recent study on 7 T MRS demonstrated increased levels of glutathione in the posterior cingulate cortex (PCC)/precuneus of patients with idiopathic generalized epilepsy compared with the healthy volunteers (2.2 ± 0.4 compared with 2.0 ± 0.2 mM/L, respectively); this controversial finding suggested increased GSH levels as an early response to oxidative stress. No difference was found in the levels of other metabolites such as GABA and glutamate [7].

- Toxic and metabolic disorders

Oxidative stress is a common mechanism underlying many toxic and metabolic disorders, leading to brain damage and cognitive impairment. Glutathione acts as a redox buffer by removing toxic metabolites, for example via GSH peroxidase. Consequently, the ratio between reduced (GSH) and oxidized (GSSG) forms of glutathione can serve as an indicator of the cellular redox state [138]. Alterations of the glutathione levels often represent a non-specific consequence of oxidative stress. However, abnormal glutathione metabolism can rarely originate from inborn errors. GSH is metabolized through the g-glutamyl cycle (Figure 1), which involves multiple enzymes. As discussed in previous sections, the synthesis of GSH relies on two consecutive steps catalyzed by γ-glutamylcysteine synthetase (γ-GCS) and GSH synthetase (GS). A deficit involving any step of the cycle or related enzymes may lead to increased oxidative stress and syndromic manifestations [138]. These manifestations can also affect the brain, such as in the case of oxoprolinuria from the GS deficit [139,140]. Oxidative stress damage may also play a pivotal role in other inborn metabolic disorders, such as mitochondrial encephalopathies. For example, overexpression of GSH in ragged red fibers is believed to represent an attempt to counterbalance the oxidative stress of Kearns−Sayre syndrome [141], a mitochondrial disorder involving the central nervous system [142,143]. Interestingly, despite oxidation-related brain damage being a well-known determinant of these metabolic disorders, data regarding the in vivo quantification of GSH in the brain is still lacking in the literature, with most evidence derived from autoptic studies [138]. Finally, a brief note on gadolinium (Gd) brain deposition is worth mentioning. This recently described phenomena consists in the accumulation of Gd salts in the deep encephalic nuclei of adult and pediatric patients after multiple administrations of gadolinium-based contrast agents (GBCA) [144,145,146], frequently used in neuroimaging. Oxidative stress may play a role in Gd ions’ toxicity, as reflected by intracellular GSH level changes [147]. In vitro studies reported Gd neurotoxicity involving the rapid accumulation of intracellular ROS and endoplasmic reticulum stress [148]. Other evidence linked Gd toxicity to impaired mitochondrial function, leading to neuron cell apoptosis [149]. A recent study on rats demonstrated that chronic GBCA exposure causes hippocampal gliosis and elevates oxidative stress and inflammation [150]. Starting from this background, one may speculate that oxidative damage is related to Gd deposition in the human brain. However, no neurological disorder has been correlated to Gd brain deposition so far, and no definite clinical sequelae have been found in patients with normal renal function [151]. The in vivo evaluation of GSH levels with MR techniques such as MRS or hyperpolarized MRI may help us better understand the pathogenesis of oxidative damage in brain disorders [152,153,154,155], paving the way for more targeted therapies and providing relevant prognostic biomarkers for future studies.

## 7. Conclusions

As a marker of oxidative stress, cerebral GSH plays a role in cell signaling, protein function, gene expression, cell differentiation, and proliferation in the brain. In recent years, several studies have analyzed the role of GSH in different neurological diseases, depicting this metabolite as a possible diagnostic biomarker and therapeutic target. In this context, MRS has become a powerful tool for the non-invasive in vivo quantification of GSH, with promising clinical applications.

## Figures and Tables

**Table 1:** Number of healthy control subjects, the corresponding age, and GSH concentration measured in the brain areas, type of scanner, method, site of voxels for the GSH measurements, and the results reported in the studies.

Ref	HC Participants	Age (Range or Mean ± SD)	Scanner	Method	Site of Detection (VOI Dimension and Brain Area)	GSH Concentration (HC)	Results
[33]	Phantoms		3 T GE + 8 channels head coil	Edited: MEGA-PRESS TR/TE = 1800/131 ms + LCModel Unedited: PRESS TR/TE = 3000/30 ms + LCModel			MEGA-PRESS appears more precise at a lower GSH concentration
[91]	Phantoms + 10 HC	26 ± 3.3	3 T Siemens + 32 channels head coil	Edited: MEGA-PRESS TR/TE = 2000/120 ms + Gannet Unedited: PRESS TR/TE = 2000/30 ms SPECIAL TR/TE = 2000/8 ms PR-STEAM TR/TE = 2000/6.5 ms + LCModel	24 cm^3^ in MFC	MEGA-PRESS 1.87 ± 0.36 mM PRESS: 1.69 ± 0.13 mM SPECIAL = 2.3 ± 027 mM PR-STEAM: 2.29 ± 0.16 mM	Reliability comparison shows more reproducible GSH measurements for unedited sequences (only for highest values, above 3 mM)
[23]	Phantoms + 5 HC	24–36; 30 ± 3	3 T Siemens + 32 channels head coil	Unedited: PRESS TR/TE = 2000/30 ms + CNN for GSH quantification	20 × 20 × 20 mm^3^ in left FC	GSH/tNAA = ~0.07–0.15	Implementation of a robust method for GSH quantification in MRS using CNN
[59]	Phantoms + 4 HC	30–45	7 T Siemens + 32 channels head coil	Unedited: 2D-COSY TR/TE = 2000/20 ms	25 × 25 × 25 mm^3^ in OC	GSH/Cr = 0.05 ± 0.01	Non-uniformly weighted sampling (NUWS) sequences produced a higher SNR
[66]	Phantoms + 13 HC	28 ± 9	3 T Magnex Scientific	Edited: Multiple Quantum Chemical Shift Imaging + Levenberg−Marquardt least square minimization algorithm	40 × 40 × 40 mm^3^ in FPC	1.2 ± 0.16 mmol/Kg	DQC filtering-based chemical shift imaging of GSH at 3T implementation
[86]	Phantoms + 6 HC	34 ± 13	3 T Siemens/Philips/GE/Canon + 32 channels head coil	Edited: MEGA-PRESS TR/TE = 2000/80 ms + Gannet	27 cm^3^ in MCC	GSH/Cr = 0.045 ± 0.013 (Philips scanner) GSH/Cr = 0.051 ± 0.007 (Siemens scanner)	In vivo GSH/Cr ratio shows relatively low variations between scanners using the universal sequence
[75]	Phantoms + 10 HC	32.6 ± 8.8	3 T Philips + 32 channels head coil	Edited: MEGA-PRESS TR/TE = 2000/120 ms MEGA-PRIAM TR/TE = 2000/120 ms + Gannet	33 × 33 × 33 mm^3^ in left and right FC	MEGA-PRESS: 2.61 ± 0.50 i.u. (left FC) 2.95 ± 0.65 i.u. (right FC) MEGA-PRIAM 2.44 ± 0.60 i.u. (left FC) 2.81 ± 0.67 i.u. (right FC)	No significant difference between MEGA-PRESS and MEGA-PRIAM in GSH estimates
[69]	Phantoms + 5 HC + simulations	31 ± 8	3 T Philips + 32 channels head coil	Edited: MEGA-PRESS TR/TE = 2000/120 ms + Gannet	36 × 36 × 36 mm^3^ in midline PC	GSH integrals normalized by the sum of the integrals from each subject averaged across all subjects ~0.4–0.5	TE of 120 ms appears to be optimal for in vivo GSH detection
[94]	Phantoms + 7 HC	23–35	3 T Philips + 8 channels head coil	Edited: MEGA-PRESS TR/TE = 2000/130 ms + AMARES Unedited: PRESS TR/TE = 2000/35 ms + jMRUI	40 × 25 × 25 mm^3^ in ACC and 30 × 30 × 30 mm^3^ in OC	MEGA-PRESS: 3.2 ± 0.6 mM (ACC) 1.4 ± 0.4 mM (OC) PRESS: 2.8 ± 0.3 mM (ACC) 2.5 ± 0.7 mM. (OC)	Physiological concentrations (<4 mM) of GSH cannot be reliably quantified from PRESS spectra at 3 T
[65]	Phantoms + 9 HC	25	4 T Varian INOVA	Edited: MEGA-PRESS TR/TE = 4000/60 ms + LCModel	30 × 30 × 30 mm^3^ in OC	1.3 ± 0.2 µmol/g	GSH concentration estimation
[25]	Phantoms + 2 HC	18–32	1.5 T Philips	Edited: DQC Unedited: PRESS	15.6–17.4 cm^3^		DQC filter for the selective in vivo detection of GSH in the human brain presentation
[84]	Phantoms + 10 HC	34.7 ± 8.8	3 T Philips + 32 channels head coil	Edited: MEGA-PRESS TR/TE = 2000/80 ms HERMES TR/TE = 2000/80 ms + Gannet	30 × 30 × 30 mm^3^ in Ins		SNR of the HERMES spectra is similar to those of MEGA-PRESS, with the benefit of saving half the acquisition time
[47]	Phantoms + 6 HC + simulations	N.D	7 T Philips	Unedited: asymmetric PRESS TE1/TE2 = 37/63 ms STEAM TR/TE = 2500/14–74 ms + LCModel	25 × 30 × 30 mm^3^ in MPFC		Optimization of the TE delays in asymmetric PRESS enables the separation of GSH without editing
[48]	Phantoms + 8 HC + simulations	32 ± 11	7 T Siemens + 32 channels head coil	Unedited: asymmetric PRESS TR/TE = 3000/3.9 ms	20 × 20 × 20 mm^3^ in MPFC and FC	GSH/tCr = 0.216 ± 0.02 (MPFC) GSH/tCr = 0.27 ± 0.03 (FC);	Glu and Gln higher in GM. GSH and Gln have a similar concentration (20–27% of Cr)
[46]	6 HC	22–26	3 T/7 T Siemens	Unedited: SPECIAL TR/TE = 4000/6 ms + LCModel	20 × 20 × 20 mm^3^ in OC	1.4 ± 0.11 mmol/Kg (3 T); 1.3 ± 0.2 mmol/Kg (7 T)	SPECIAL with ultrashort TEs resulted in a high SNR and allow to reduce RF power requirements and improve chemical shift displacement errors
[56]	15 HC	24.9 ± 3.5	3 T Siemens + 32 channels head coil	Edited: MEGA-PRESS TR/TE = 3200/68 ms + LCModel Unedited: SPECIAL TR/TE = 3200/8.5 ms + LCModel	30 × 30 × 20 mm^3^ in DLPC and M1	MEGA-PRESS: 0.5–3 mmol/L (M1) 3–4 mmol/L (DLPC) SPECIAL: 1.3–2.4 mmol/L (M1 and DLPC)	GSH levels detected with reasonably good precision using SPECIAL, but poor precision using MEGA-PRESS
[55]	21 HC	32.2 ± 8.1	3 T Siemens + quadrature head coil	Unedited: SPECIAL TR/TE = 3000/6 ms + LCModel	15 × 15 × 15 mm^3^ in left A	1.03 ± 0.38 mmol/L (CRLBs: 24 ± 11 only in 16/21 HC)	Only in a small portion of the acquired spectra GSH passed the CRLB threshold of 20%
[43]	18 HC	N.D.	3 T Siemens	Unedited: PRESS TR/TE = 2000/30 ms + LCModel	25 × 25 × 15 mm^3^ in SMA	~2.2–2.6 mmol/Kg	No difference in GSH concentration recorded between HC and PSP
[90]	22 HC	12–14	3 T Siemens	Unedited: 2D J-resolved PRESS TR/TE = 2000/22 ms + LCModel	20 × 20 × 30 mm^3^ in RACC		GSH variation factor results of 8.6 ± 4.1%, significant Pearson correlation (0.821) resulted between test and retest
[152]	63 HC	40–60	3 T Siemens	Unedited: 2D J-resolved MRS TR/TE = 2000/31–229 ms + ProFit	19 cm^3^ in RACC	GSH/H20 = 0.003–0.004	GSH significantly increased for HC receiving supplements when compared with the placebo
[9]	5 HC	32 ± 8	7 T Agilent + 8 channels head coil	Edited: JDE semi-LASER TR/TE = 3200/72 ms + LCModel Unedited: STEAM TR/TE = 3000/10 ms + LCModel	30 × 30 × 30 mm^3^ for JDE semi-LASER and 20 × 20 × 20 mm^3^ for STEAM in midline OC	1.34 ± 0.13 mM (JDE semi-LASER) 2.15 ± 0.16 mM (STEAM)	Better reliability results (in terms of Coefficient of variation CV) for JDE semi-LASER when compared to STEAM
[45]	21 HC	Neonates	1.5 T GE	Unedited: PRESS TR/TE = 3000/20 ms + LCModel	29 × 10 × 11 mm^3^ in WM; 11 × 24 × 11 in Th; 22 × 13 × 15 in GM	2.1 ± 0.7 mmol/Kg (WM) 2.4 ± 0.8 mmol/Kg (Th) 2.5 ± 0.5 mmol/Kg (GM)	Absolute brain GSH content in premature infants at term was not considerably different from that in fullterm infants
[35]	5 HC	25–32	3 T Siemens + quadrature head coil	Edited: DQF TR/TE = 3000/70 ms	30 × 30 × 30 mm^3^ in left and right PC	0.91 ± 0.16 mM (left PC) 0.89 ± 0.16 mM (right PC)	Sequence shown to be invariant to phase difference between excitation and DQF generating pulse.
[51]	10 HC	26.1 ± 9	3 T Siemens	Unedited: STEAM TR/TE = 2000/6.5 ms + LCModel	6 cm^3^ in ACC and PCC	2.74 ± 0.2 i.u. (ACC) 2.07 ± 0.0025 i.u. (PCC)	Good reliability results in terms of coefficient of variation CV (<10%)
[153]	60 HC	60–85	3 T Siemens	Edited: Multiple Quantum Chemical Shift Imaging + Levenberg−Marquardt least square minimization algorithm	50 × 50 × 30 mm^3^ in FC and PC	1.27 ± 0.32 mmol/Kg (FC) 1.28 ± 0.27 mmol/Kg (PC)	glutathione concentrations in brain regions were positively correlated with milk servings
[85]	18 HC	Neonates	3 T Philips	Edited: HERMES TRT/E = 2000/80 ms + Gannet	31 × 25 × 20 mm^3^ in Th and ACC	0.55–0.7 i.u. (ACC) 0.5–0.58 i.u (Th)	lower GSH levels in Th compared to the ACC and higher GSH levels in the ACC following tissue-correction
[87]	20 HC	21–35; 29 ± 5	3 T Philips + 32 channel head coil	Edited: HERMES TRT/E = 2000/80 ms + LCModel	25 × 25 × 25 mm^3^ in MACC	GSH/tCr = 0.18 ± 0.04	HERMES showed to be more sensitive to motion, as variability of spectral quality measures were observed for GSH when only retrospective outlier removal was applied.
[81]	40 HC		3 T Philips	Edited: HERMES TRT/E = 2000/80 ms + Gannet	Ranging from 30 × 30 × 30 to 36 × 36 × 36 mm^3^ in medial PC		The multi step Frequency and Phase Correction approach (msFPC) results in improved correction of frequency/phase errors in multiplexed GABA-/GSH-edited MRS experiments.
[72]	67 HC	8–12	3 T Philips	Edited: HERMES TR/TE = 2000/80 ms + Gannet	30 × 30 × 30 mm^3^ in right SM, SMA, and right Ins	0.56 ± 0.14 i.u. (SM) 0.57 ± 0.15 i.u. (SMA) 0.69 ± 0.19 i.u. (Ins)	Robust Spectral Registration (rSR) reduced more subtraction artifacts than the multistep method
[93]	12 HC	25 ± 2.5	3 T Siemens + 32 channel head coil	Edited:MEGA-PRESS TR/TE = 2000/120 ms HERMES TRT/E = 2000/80 ms + Gannet	30 × 25 × 25 mm^3^ in DACC	1.96 ± 0.49 i.u. (MEGA-PRESS) 3.95 ± 0.44 i.u. (HERMES)	MEGA-PRESS provide more reproducible GSH (in terms of CV%) quantification compared to HERMES
[73]	4 HC	47.3 ± 5.6	3 T GE	Edited: MEGA-PRESS TR/TE = 2000/80 ms	30 × 30 × 30 mm^3^ in PC	2 mM	Phantoms confirm GSH MEGA-PRESS signal and that GSSG would be undetectable at concentrations expected in vivo
[82]	9 HC	23	4 T Varian INOVA	Edited: DWE with MEGA-PRESS TR/TE = 4500/112 ms + LCModel	30 × 30 × 30 mm^3^ in midsagittal OC	0.8 ± 0.1 µmol/g	Double editing did not compromise sensitivity
[3]	44 HC (22 young + 22 elderly)	Young = 20.4 ± 1.4 Elderly = 76.6 ± 6.1	4 T Varian INOVA	Edited: DWE with MEGA-PRESS TR/TE = 4500/122 ms + LCModel	30 × 30 × 30 mm^3^ in midsagittal OC	Young = 0.31 ± 0.05 i.u. Elderly = 0.20 ± 0.08 i.u.	Elderly subjects had a lower GSH concentration than younger subjects
[83]	12 HC		4 T Varian INOVA	Edited: DWE with MEGA-PRESS TR/TE = 4500/102 ms + LCModel	30 × 30 × 30 mm^3^ in OC	0.7–0.9 µmol/g	GSH concentration remains costant after intravenous vitamin C infusion
[67]	11 HC	61.5 ± 10.5	3 T GE + 8 channels head coil	Edited: MEGA-PRESS TR/TE = 1500/68 ms + in-house software developed in MATLAB	20 × 25 × 25 mm^3^ in PG and MC	GSH/W = 1.6 ± 0.4 × 10^−3^ i.u. (MC)	Significantly lower GSH in ALS patients when compared with HC
[77]	11 HC	30 ± 11	3 T Philips	Edited: MEGA-PRESS TR/TE = 2000/131 ms	50 × 30 × 30 mm^3^ in PC	1.20 ± 0.14 mM	Optimal TE = 130 ms. Stroke patients not significantly different from HC
[154]	10 HC	18–65	3 T	Edited: MEGA-PRESS TR/TE = 1500/68 ms	30 × 30 × 20 mm^3^ in OC		Anhedonia and GSH negatively correlated
[155]	13 HC	18–45	3 T GE	Edited: MEGA-PRESS TR/TE = 1500/68 ms	30 × 30 × 20 mm^3^ in OC		No differences between HC and CFS patients
[96]	44 HC (25 males and 19 females)	23.6 ± 2.1	3 T Philips	Edited: MEGA-PRESS TR/TE = 2500/120 ms	2.5 cm^3^ in FC PC, Hyp and C	~20–22 a.u. (FC females) ~15–22 a.u. (FC males) ~30 a.u. (PC females) ~17–25 a.u. (PC males) ~15 a.u. (Hyp females) ~15 a.u. (Hyp males) ~14–17 a.u. (C females) ~10–15 a.u. (C males)	Higher GSH in young, gender matched parietal cortex hippocampus vs. older patients
[10]	21 HC	65 ± 5	3 T Philips	Edited: MEGA-PRESS TR/TE = 2500/120 ms + KALPANA	15–16 cm^3^ in FP Hyp	1.12 ± 0.18 mmol/L (FC) 1.02 ± 0.17 mmol/L (Hyp)	Significant reductions in GSH in both the frontal cortex and hippocampus in disease
[97]	17 HC	38.8 ± 13.1	3 T GE	Edited: MEGA-PRESS TR/TE = 1800/68 ms + LCModel	25 × 40 × 30 mm^3^ in DLPC 28 × 30 × 25 mm^3^ in ACC	GSH/Cr = 0.11 ± 0.03 (ACC) GSH/Cr = 0.11 ± 0.03 (left DLPC);	Higher GSH in patients
[74]	16 HC	21–41; 30 ± 7.2	3 T GE + standard quadrature coil	Edited: MEGA-PRESS TR/TE = 1500/94 ms + GE software	28 × 30 × 22 mm^3^ in PMPC	0.928 ± 0.24 mM	No significant differences between GSH concentration of HC and patients
[3]	14 HC	32 ± 10	7 T Magnex Scientific	Unedited: STEAM TR/TE = 5000/8 ms + LCModel	Ranging from 6 × 6 × 13 to 20 × 20 × 20 mm^3^ in FWM, LS, PCC, OC, P, SN, and CV	Ranging from 0.50 ± 0.1 μmol/g (OC) to 1.2 ± 0.2 μmol/g (CV)	Lower GSH concentration in elderly subjects than in younger subjects
[89]	10 HC	25 ± 3	7 T Philips + 16 channels head coil	Unedited: STEAM TR/TE = 3000/15 ms + LCModel	20 × 20 × 20 mm^3^ in OC	2.28 ± 0.1 µmol/g	GSH increased during visual stimulation
[50]	10 HC	20 ± 3	4 T Varian INOVA	Edited: MEGA-PRESS TR/TE = 4500/68 ms + LCModel Unedited: STEAM TR/TE = 4500/5 ms + LCModel	17 cm^3^ in ACC and 8 cm^3^ in OC	1.6 ± 0.4 µmol/g (ACC) 1.6 ± 0.2 µmol/g (OC)	Validation of glutathione quantitation from the STEAM spectra
[7]	10 HC	20–70; 39.2 ± 15.3	7 T Siemens + 32 channels head coil	STEAM TR/TE = 8500 (9 subjects)−9300 (1 subject)/6 ms + LCModel	20 × 20 × 20 mm^3^ in PCC/precuneus	1.9 ± 0.3 mmol/L	GSH levels higher in IGE (idiopathic generalized epilepsy) compared with HC
[137]	8 HC	19–53; 28.4 ± 10.7	1.5 T Philips + birdcage head coil	PRESS + DCQ (double quantum coherence) filtering	25 × 25 × 25 cm^3^ POC	GSH/H_2_O = 2.3 ± 0.9 × 10^−5^ (right POC) 2.5 ± 1.2 × 10^−5^ (left POC)	GSH/water ratio significantly reduced in both hemisphere Ins epileptic patients compared with HC
[44]	7 HC	6–17	3 T Siemens + 32 channels head coil	PRESS TR/TE = 1980/30 ms + LCModel	variable from 3 to 8 cm^3^ in the right gangliocapsular region	2.0 ± 0.5 mM	Higher levels of brain GSH in KD patients compared with HC
[68]	17 HC		7 T and 3 T Siemens + 16 channels head coil (7 T)	MEGA-PRESS TR/TE = 2000/68 ms	3.5 × 2.5 × 2.3 cm^3^ in left or right M1 (3 T and 7 T) and pons (3 T)		No significative difference in brain GSH between ALS patients and HC using 3 T scanner
[67]	11 HC	58.5 ± 6.6	3 T GE + 8 channels head coil	PRESS with J-edited spin echo method TR/TE 1500/68 ms	single voxel on primary motor cortex (M1)	GSH/H_2_O = 1.6 ± 0.5 × 10^−3^ GSH/Cr 1.9 ± 0.8 × 10^−2^	Reduced GSH in ALS patients compared with HC
[131]	15 HC	55–75	3 T GE + 8 channels head coil	PRESS with J-edited spin echo method TR/TE 1500/68 ms	2.5 × 2.5 × 2.5 cm^3^ PCC and precuneus		GSH reduction with increased levels of amyloidosis
[96]	85 HC	males 26.4 ± 3.0; females 23.6 ± 2.1	3 T Philips + 32 channels head coil	MEGA-PRESS TR/TE = 2500/120 ms	2.5 × 2.5 × 2.5 cm^3^ in several brain regions		Female HC have higher GSH levels compared to male HC with a specific distribution pattern
[128]	29 HC	18–50	3 T GE	PRESS TR/TE = 3000/30 ms + LCM model	20 × 20 × 15 mm^3^ in BG and 16 × 24 × 20 mm^3^ in DMPFC	2–3 mM (DMPFC and BG)	No difference between GSH levels in ASD patients and HC
[99]	40 HC	18–30	3 T Philips + 32 channels head coil	MEGA-PRESS TR/TE = 2048/68 ms	3 × 3 × 3 cm^3^ in five different regions (OC, left/right MT, TC, and PC)	Occipital 6.91 (0.68) i.u. Left MT+ 5.51 (0.86) i.u. Right MT+ 6.59 (0.67) i.u. Temporal 7.17 (0.93) i.u. Parietal 5.17 (0.59) i.u	No difference in GLX metabolites between ASD patients and HC
[15]	12 HC	50–84; 61.5 ± 4.9	3 T GE + 8 channel head coil	PRESS TR/TE = 2000/35 ms + LCModel	20 × 20 × 20 mm^3^ in ACC	GSH/Cr = 0.22 ± 0.06	Increased GSH in patients with depressive symptoms
[62]	17 HC	20–29	3 T GE + 8 channel head coil	PRESS TR/TE = 2000/35 ms + LCModel	20 × 20 × 20 mm^3^ in ACC	1.47 ± 0.47 i.u.	Less GSH in the ACC of patients with high risk of alcohol abuse
[14]	49 HC	18–30	3 T GE + 8 channel head coil	PRESS TR/TE = 2000/35 ms + LCModel	20 × 20 × 20 mm^3^ in ACC and 1.5 × 3.0 × 1.0 in left Hyp		Decreased ACC-GSH with tobacco use in patients with bipolar disorder. No differences in GSH levels with alcohol use
[61]	25 HC		3 T GE + 8 channel head coil	PRESS TR/TE = 2000/35 ms + LCModel	320 × 20 × 20 mm^3^ in ACC		Distinct neurometabolic profiles are evident in young people with major psychiatric disorders
[24]	45 HC		7 T Philips	STEAM TR/TE = 2000/17 ms + LCModel	20 × 18 × 25 mm^3^ in ACC, 40 × 12 × 18 mm^3^ in left Ins, 20 × 22 × 20 mm^3^ in OC	1.75 ± 0.31 mM (ACC) 1.72 ± 0.20 mM (left Ins) 1.5 ± 0.17 mM (OC)	Reduced GSH in ACC of patients with schizophrenia
[127]	25 HC	34.0 ± 12.3	3 T Siemens + 32 channels head coil	2DJ PRESS	20 × 20 × 20 mm^3^ in PCC	GSH/Cr = 0.25	Lower GSH/Cr in PCC of patients with obsessive compulsive disorder
[95]	26 HC	22.77 ± 4.05	3 T GE + 8 channel head coil	MEGA-PRESS TR/TE = 1500/68 ms	20 × 40 × 30 mm^3^ in MPFC	GSH/H_2_O = 0.0015–0.0018	No difference in GSH levels between HC and patients at a clinical high risk for psychosis
[98]	9 HC	22.56 ± 2.35	3 T GE + 8 channel head coil	MEGA-PRESS TR/TE = 1500/68 ms	4.5 × 2.5 × 2.5 mm^3^ in striatum	GSH/H_2_O = 1.10 ± 0.10 × 10^−3^	Striatal GSH deficit in patients with a first episode of psychosis
[97]	17 HC	40.4 ± 12.3	3 T GE + 8 channel head coil	MEGA-PRESS TR/TE = 1800/68 ms + LCModel	28 × 30 × 25 mm^3^ in ACC and 25 × 40 × 30 mm^3^ in DPLFC	GSH/Cr = 0.11 ± 0.03 (ACC)	Higher GSH levels in PTSD patients
[42]	41 HC	56–80; 68.7 ± 5.8	3 T GE + 8 channel head coil	PRESS TR/TE = 2000/35 ms + LCModel	20 × 20 × 20 mm^3^ in ACC		Elevated ratios of GSH in subjects with mild cognitive impairment
[126]	18 HC	15–29	3 T GE	PRESS TE 30 ms + LCModel	2 cm in both TC	1.5–2 mM	GSH levels significantly higher in patients with a first episode of psychosis

## Abbreviation

HC = Healthy Controls;PSP = Progressive Supranuclear Palsy; ALS = Amyotrophic Lateral Sclerosis; ASD = Autism Spectrum Disorder; Chronic fatigue syndrome CFS; CNN = Convolutional Neural Network; DQC = Duble Quantum Coherence; DQF= Double Quantum Filter; DWE = Double Editing; ACC = Anterior Cingulate Cortex; FC = Frontal Cortex; MFC = Medial Frontal Cortex; OC = Occipital Cortex; FPC = Fronto Parietal Cortex; MCC = Middle Cingulate Cortex; PC = Parietal Cortex; Ins = Insula; MPFC = Medial Pre-Frontal Cortex; DLPC = Dorso-Lateral Prefrontal Cortex; M1 = Primary Motor Cortex; A = Amygdala; SMA = Supplementary Motor Area; RACC = Rostral ACC; WM = White Matter; GM = Grey Matter; Th = Thalamus; PCC = Posterior Cingulate Cortex; MACC = Medial ACC; MPC = Medial Parietal Cortex; SM = Sensorimotor; DACC = Dorsal ACC; PG = Precentral Gyrus; MC = Motor Cortex; Hyp = Hyppocampus; C = Cerebellum; PMPC = Posterior Medial Prefrontal Cortex, FWM = Frontal WM; LS = Limbic System; P = Putamen; SN = Substantia Nigra; CV = Cerebellam Vermis; DMPFC = dorso-medial prefrontal cortex; BG = Basal Ganglia; POC = Parieto-Occipital Cortex; TC = Temporal Cortex; MRS = Magnetic Resonance Spectroscopy.
